# Intentional Tooth Replantation: Current Evidence and Future Research Directions for Case Selection, Extraction Approaches, and Post-Operative Management

**DOI:** 10.3390/dj14010059

**Published:** 2026-01-15

**Authors:** Rahul Minesh Shah, Thomas Manders, Georgios Romanos

**Affiliations:** Department of Periodontics and Endodontics, School of Dental Medicine, State University of New York at Stony Brook, Stony Brook, NY 11794, USA; rahul.shah@stonybrookmedicine.edu (R.M.S.);

**Keywords:** endodontic therapy, atraumatic extraction, intentional tooth replantation, root morphology

## Abstract

**Background:** Intentional tooth replantation (ITR) is a promising treatment option for preserving teeth in cases where conventional endodontic therapy is challenging, or when previous endodontic treatment and apicoectomy have been unsuccessful. The procedure involves extracting the compromised tooth, preserving the alveolar socket and root surface, performing extraoral endodontic therapy, and replanting the tooth in the alveolar socket. **Objective:** An increase in evidence-based support for ITR has improved the viability of ITR as a treatment option for patients. This review aims to further establish and provide new areas of potential research for ITR with respect to root morphology, extraction, and surgical techniques, maintenance of the tooth socket, and methods for post-op stabilization. **Materials and Methods:** A literature review was performed across PubMed from 1 January 1980 to 1 July 2025, with a focus on oral surgery techniques, atraumatic extraction techniques, topographical discrepancies in root system anatomy, and ITR procedural outcomes. **Conclusions:** Although ITR is not a common procedure performed in contemporary clinical practice, gathering sufficient data on the variables influencing the procedure may help patient outcome and improve communication between the endodontist and oral surgeons.

## 1. Introduction

Intentional tooth replantation (ITR) has become a promising clinical technique for saving a patient’s tooth. Over the past decades, the ITR success rate has increased, and its procedural steps have been continuously refined with the development of new materials and clinical protocols. A recent consensus study published recently in the International Journal of Oral Science illustrated the variations in clinical protocols, biological aspects, and importance of establishing evidence-based procedural guidelines [1].

In most cases, prior to ITR, multiple steps have been taken to address initial infection, recurrent infection, pulpal pathology, and/or peri-radicular pathology. Typically, patients will undergo non-surgical RCT to remove infection and pain. Non-surgical RCT treatment is relatively consistent in terms of its success rate, at 92.6% [2]. Although success rates are similar in both anterior and posterior dentition, in some cases there is a limited field of view when accessing, nuance in anatomy, furcation involvement, and complex root system morphology which can all lead to possible complications [3,4]. When non-surgical RCT and non-surgical retreatment fails, clinicians may select endo microsurgery as the next course of action. Endo microsurgery aims to prevent bacterial leakage from the root canal system into the peri-radicular tissue through removal of the compromised root end and placing a root-end filling [5]. Like non-surgical RCT, limitations do exist. Access and the ability to visualize the apices of the teeth in question may be difficult. For example, thick cortical bone in the posterior mandibular area can make root end visualization difficult. This can lead to complications, such as an inability to prepare a window opening parallel to the root end, as well as a greater chance of perforation of the root [6]. Complications involving vital structures, such as the inferior alveolar nerve and maxillary sinus are also more likely when performing apical surgery. Thus, ITR may be useful in situations where conventional retreatment is hindered by unfavorable tooth positioning or restricted access to the root canal system.

Removal of the tooth from its alveolar socket and allowing the clinician to gain better access and visualization of the endodontic system may increase the chance of survival for the tooth post-replantation. The American Association of Endodontics (AAE) reports that ITR has an 89.1% survival rate on par with conventional endodontic therapies, such as RCT and endodontic microsurgery [2,7]. From a financial perspective, ITR serves as a practical option when compared to traditional single implant placement. A meta-analysis showed that the cost of ITR could be as much as one-fourth the cost of an implant procedure, which does not include the opportunity cost of time spent at multiple appointments for implant placement and restorations [8].

There is agreement with respect to certain aspects of the procedure such as preserving periodontal ligament (PDL) fibers, minimizing extraoral treatment time, and being selective with respect to root anatomy and anatomical structures [9]. The AAE indicates the procedure is a viable solution for teeth that are difficult to access through endodontic surgery as well as teeth that have proximity to vital structures. Procedurally, the AAE recommends atraumatic extraction of the tooth, minimizing extraoral treatment time, and splinting only if necessary [10]. The European Society of Endodontology outlines similar procedural guidelines emphasizing importance of PDL preservation, removal of granulation tissue, careful extra-alveolar management of the tooth, and adequate post-operative care [11]. While these are widely accepted clinical protocols, extraction techniques, such as flap elevation, sectioning of root systems, and conventional extraction modalities have not been discussed in the literature and are areas that need further research to expand the procedure’s scope.

In this review, morphological constraints from varying geographical regions were investigated to provide evidence for which teeth would be most viable for ITR. Extraction techniques were examined and introduced as areas of potential research for ITR of teeth that may require advanced surgery to preserve the socket, root surface, and root apex. Finally, post-operational treatment was investigated [12]. The data and potential techniques presented here may allow oral surgeons and endodontist to determine how to best proceed with ITR and improve interdisciplinary collaboration.

## 2. Materials and Methods

A structured literature search conducted by the authors for each topic was conducted individually. Given the authors’ backgrounds in both endodontics and oral surgery, the authors were able to use their expertise to accurately analyze and select articles pertaining to the topics. These topics were morphological variations in root canal systems, extraction techniques, socket management, and PDL preservation. Each search was performed in the database PubMed from 1 January 1980 to 1 July 2025, and Boolean operator “+” and “or” were used as well as “,” to separate key words. Abstracts and titles were manually reviewed and assessed to determine if they met the inclusion criteria of the current study. Articles that did not pertain to the topic of intentional tooth replantation or did not provide any information to further develop an understanding of the procedure were excluded. Zotero Citation software (v 6.0.37; Corporation of Digital Scholarship, Vienna, VA, USA) was used for reference management.

### 2.1. Inclusion Criteria

Articles were included based on the following criteria:

- Keywords: intentional tooth replantation, tooth auto transplantation, apicoectomy, endodontic microsurgery, extraction, flap elevation, sectioning, socket management, furcation management, root surface treatment, interradicular bone preservation
- Time period: 1 January 1980–1 July 2025
- Articles, more specifically case reports and human clinical trials/case studies, that pertained to root anatomy, root canal anatomy, oral surgery techniques, atraumatic extraction techniques, geographical discrepancies in root systems, PDL preservation, and splinting

### 2.2. Exclusion Criteria

Articles were excluded based on the following criteria:

- Articles in languages other than English
- Animal studies and in vitro studies
- Studies that were not related to the topic at hand (for example, avulsed teeth due to trauma were excluded when discussing post op stability)

**Figure 1:**
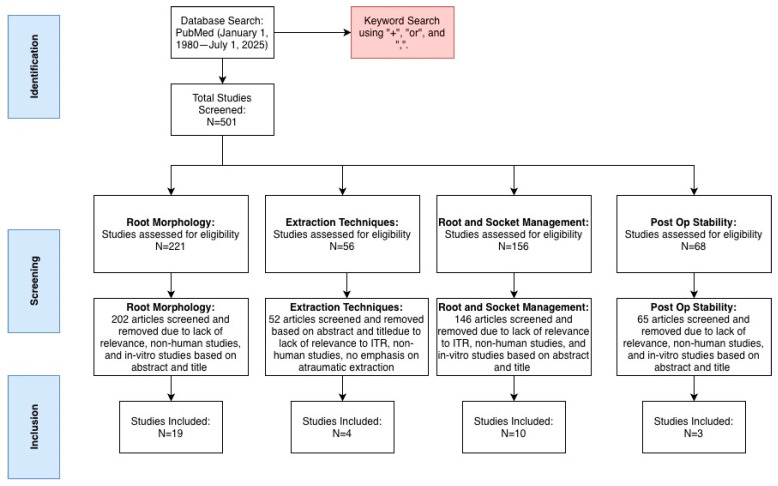
Schematic representing literature search and respective identification, screening, and inclusion steps.

Figure 1 outlines the literature search process.

With regard to morphological variations in root canal systems, 221 articles were identified. After screening and eliminating articles that were in vitro studies and studies that did not pertain to root or root canal anatomy of human dentition, 19 articles remained. These articles were analyzed and used for discussing geographical variations in root and root canal morphology.

With regard to extraction techniques, 56 articles were identified using key words atraumatic extraction, flap elevation, and tooth sectioning. Upon review, 4 articles were selected to discuss these techniques as they pertain to ITR.

With regard to socket management, PDL preservation, and root surface treatment, 156 articles were identified. Upon review, 10 articles were selected for further discussion based on their relevance to ITR and exclusion criteria.

**Figure 2:**
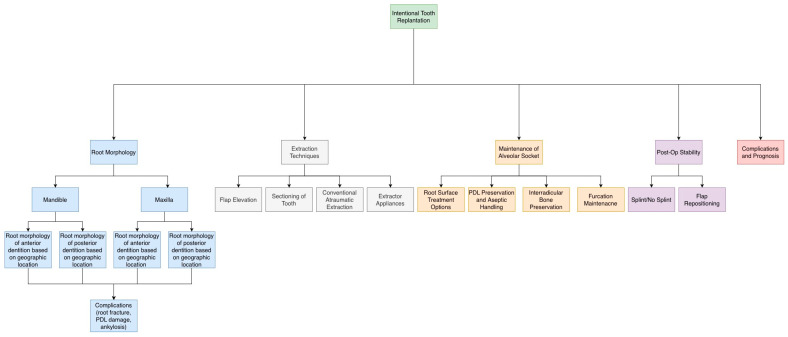
Schematic representing ITR procedural aspects that clinicians should consider when performing surgery, endodontic therapy, and replantation.

With regard to post-operative stability, 68 articles were identified using stability and tooth replantation as key words. Upon review, 3 articles were selected for further discussion based on relevance to ITR and exclusion criteria. Figure 2 outlines the aspects of the ITR procedure that will be discussed

## 3. Morphological Variations in Root and Root Canal Systems

Highlighted by Frank Vertucci in 1984, the root canal anatomy of permanent teeth can have varying characteristics with variable frequencies in the number of canals and anatomical positioning of canals [13,14]. Root morphology, in addition to their canals, may vary by age, demographics, and genetics and are essential in determining the viability of an ITR procedure [1]. Herein, we present morphological characteristics of root and root canal systems that vary by population, demographic, and area of dentition. Ideally, for ITR, a straight conical root system is desired with minimal deflection such that the tooth can be extracted atraumatically without damage to the root surface or PDL fibers [1]. Table 1, Table 2, Table 3 and Table 4 show different morphological characteristics for the anterior and posterior mandibular and maxillary dentition based on geographical location.

The mandibular anterior region presents with similarities in terms of the number of roots, but exact anatomical form may vary among populations. Studies in these tables demonstrate that for mandibular incisors, most people will have one root with a low prevalence of dilaceration. However, this may vary based on geographic location and should be confirmed with modalities such as radiographs and CBCT. For example, populations from China may have root furcations in their mandibular canines and lateral incisors, which may make it more difficult to extract the tooth with little rotational and torsional manipulation possibly compromising the integrity of the root surface and cortical plates [19].

Dilacerations can make ITR difficult due to the inability to atraumatically extract and possible requirement of flap elevation [23]. Though the distribution of dilacerated teeth may vary by population, maxillary lateral incisors and mandibular canines are more likely to be unfavorable for an ITR procedure due to their higher prevalence of curvature [17,24]. On the other hand, mandibular and maxillary central incisors, as well as mandibular lateral incisors, may be more favorable based on limited root curvature [17,22]. Studies have shown that root fractures ranged from 5% to 7% for all extractions involving dilacerated roots indicating the need for caution when conducting ITR on these teeth [34].

The maxillary premolar teeth present unique challenges due to their proximity to vital structures like the maxillary sinus, as well as their typically longer roots and higher incidence of dilaceration, as shown in Table 2. The average distance from the maxillary sinus to the root tip of premolars in the Chinese population was 2.47 ± 3.45 mm indicating the technique needed for extraction becomes more advanced during ITR [35]. The same can be said for the mandibular dentition and its proximity to the inferior alveolar nerve, with an average distance of 9.8 ± 3.0 mm from the alveolar crest [36]. Population studies from China have found root lengths for mandibular molars in the 9–10 mm range, indicating a more difficult extraction if ITR were to be performed [29]. However, if clinicians are trying to save a tooth due to apical pathology close to vital structures, ITR can eliminate working in vital structure zones directly by careful extraction of the tooth.

In terms of posterior dentition morphology, the complexity involved with extractions increases and requires more intricate planning. Both mandibular and maxillary premolars and molars can vary in the number of roots they have, typically ranging from 1 to 2 roots in premolars and 1 to 4 roots in molars [27,29,30]. As the number of roots increases, the potential risk for fracture within the tooth increases due to the asymmetric nature of roots, divergence, and the need for advanced operator skill [37]. A fractured tooth post extraction would eliminate the chance of ITR working for the patient. Some populations, such as those from Colombia, may fare better with molar extractions during ITR given their high prevalence of root fusion and conical structure [1,31]. Root fusion results in a conical tooth structure and less traumatic extraction, preventing damage to the PDL fibers and root surface. Studies in Table 3 and Table 4 show a higher likelihood of having angulated roots in almost all populations for posterior dentition highlighting the importance of a highly skilled operator and adequate understanding of root morphology for ITR in this area.

While morphological constraints and risk of traumatic extraction are a major consideration for ITR, periodontal disease is a factor that must be considered. Cho et al. studied the success rates for ITR for periodontally compromised teeth with at least 1 pocket depth greater than 6 mm [38]. It was found that patients who are older and have an increased number of pocket depths are more prone to the ITR procedure failing. Therefore, while root morphology is one of the most important factors for ITR, a comprehensive decision must be made considering the patient’s entire dental workup.

## 4. Extraction Techniques and Their Limitations Within ITR

As with all dental extractions, assessment of the patient’s systemic status, need for antibiotic prophylaxis, and the preservation of a sterile surgical field are key. Lin et al. and the ESE advise antibiotic prophylaxis only for patients who require it for medical conditions such as artificial heart valves, infective endocarditis, after joint surgeries, etc. [1,11]. While there are no direct systemic contraindications to conducting ITR procedure, the entire medical history of the patient must be considered before surgery. Additionally, it is recommended that the surgical site be void of any supragingival calculus and disinfected with 0.12% chlorohexidine to reduce risk of post-operative infection [1,11].

The ultimate success of the ITR procedure hinges on factors such as preservation of PDL, limiting trauma to the tooth surface, and maintaining the alveolar socket [1]. Due to these requirements, the use of conventional extraction techniques with elevators, luxators, and periotomes is contraindicated because of direct leverage forces causing damage to the root surface. The use of forceps alone is recommended with the beaks positioned above the cementoenamel junction, thereby avoiding contact with the cementum and root surface [1]. Varying atraumatic extraction techniques have been used for ITR, such as prior orthodontic extrusion, wheel shaft mechanisms, and disrupting the PDL before the procedure [1]. However, these techniques may require increased time in the chair and discomfort for the patient.

Flap elevation allows for better visualization and allows for a more accessible extraction, but it may be contraindicated during ITR procedures if destruction of cortical bone and PDL fibers occurs [39]. During flap elevation, bone from the cortical plate may need to be removed in some cases. This would effectively remove any periodontal ligament connection and increase the risk of alveolar bone resorption during bone remodeling in the future [40]. If these invasive flap elevation procedures are needed to effectively remove the tooth, ITR would not be a suitable option. However, there have been studies where teeth have been extracted and intentionally replanted through elevation of a mucogingival flap without damage to the periodontal membrane on the root surface [41]. The teeth were taken out with forceps only. Consequently, flap elevation may be beneficial for visualization and proper root crown alignment without compromising the ability to replant successfully. Additionally, when working near important anatomical structures, mucogingival flap elevation may help with visualization of the surgical area. Hence, there may be some instances where flap elevation can be beneficial for the extraction of the tooth in ITR.

A method of extraction that has not been reported within the scope of ITR is tooth sectioning of multirooted teeth. Sectioning of the tooth makes the removal of the tooth easier which would be beneficial in ITR. An example of this with the intent of preservation is bicuspidization. This is performed by bisecting a molar tooth into two units of single bicuspid molars to help the patient properly maintain the area [42]. These single bicuspid molars are then endodontically treated and restored with an indirect restoration. The process of bicuspidization usually involves periodontally compromised teeth with furcation involvement [42]. Through full-thickness flap elevation and a vertical cut given at the center of the tooth, the tooth can be separated at the furcation. This technique may sound promising, but to make the correct section, visualization of the furcation is necessary, which would not be possible without cortical plate bone removal in that area. For ITR, sectioning or bicuspidization may be used for teeth that are periodontally compromised and have good visualization without bone removal. This will allow for the tooth to be atraumatically extracted and subsequently replanted in their respective sockets. Although the procedure has not been performed before for ITR, it is an area that should be examined for increasing the scope of the procedure.

Anterior teeth, given that their root anatomy and curvature are not complex, benefit more from minimal rotation and extracting the tooth along the long axis of the tooth. New minimally invasive extraction methods, such as vertical tooth extraction (85.4% success rate), should also be considered [41]. These may include uses of pull ropes and extractor appliances that can extract by incrementally increasing the traction force in the appliance, slowly pulling the tooth out in one axis without luxation or torsional forces. This extraction technique within the context of ITR resulted in an incidence rate of 9.7% with respect to post-operative complications [1]. Further research needs to be performed on the effectiveness of these systems, but they remain a promising option for operators carrying out ITR.

## 5. Cleaning and Debridement of Root and Socket

Following the extraction of the tooth, the importance of maintaining the socket, furcation, and root surface is key to a long-term favorable outcome [1]. Aseptic handling and reducing bacterial contamination while the tooth is treated extraorally must be emphasized to reduce the risk of infection and improve healing outcomes following replantation. The use of a sterile wet gauze saturated with Hank’s balanced salt solution, sterile gloved fingers, or forceps can be used to accomplish this [43]. All extraoral procedures should be performed in a timely manner as Lin et al. recommends that the extracted tooth be replanted within 10–15 min to limit PDL necrosis following extraction [1,44]. The consensus on tooth replantation highlights gentle curettage, removing granulomatous tissue and cysts within the alveolar socket, and restricting any manipulation to the root surface of the tooth. It is also important to ensure that no fractures or perforations are present, and the furcation area remains intact. There is general acceptance of these techniques in ITR, but areas regarding interradicular bone preservation, treatment of the root surface, and furcation management have been limited in their discussion.

Interradicular bone serves a crucial role in promoting the stability of the tooth after replantation [45]. Following a molar extraction, the primary areas of bone available are the periphery of the socket and the interradicular septal bone. Areas where there are insufficient levels of septal bone due to the extraction technique or compromised bone levels in the patient may not offer stability when replanting the tooth. Thus, preservation of bone in these areas is key to stability of the tooth and reattachment of PDL fibers. There are no cases where bone grafting was used to help preserve these areas due to periodontally compromised defects or loss of bone during extraction in ITR. However, there have been cases of ITR where other treatment modalities have been used to improve the stability of the tooth. ITR of a periodontally compromised mandibular central incisor with significant bone loss was carried out. During the procedure, platelet-rich plasma, in combination with bioactive glass graft material and non-resorbable membrane, was used, resulting in adequate bone fill one-year post-procedure [46]. Another study involving a mandibular second premolar with a periapical cyst was selected for ITR with the use of a concentrated growth factor membrane placed in the alveolar fossa [47]. Thirteen months following ITR, the patient presented with no cyst and new bone formation in the apical region. These case reports feature the use of regenerative dentistry methods to improve bone fill and increase the stability of the replanted tooth. More research and studies need to be conducted to evaluate the long-term efficacy of these techniques, but they may be promising in preserving interradicular areas.

Treatment of the root surface is just as important as maintaining the interradicular bone in the alveolar socket. The consensus on ITR cautions against directly scraping the root surface to preserve periodontal cells and cementum [1]. The use of a liquid medium such as Hank’s balanced salt solution, electrolyte solutions, or milk is also recommended to maintain the activity of the PDL [1]. Several studies have introduced root surface treatments that are beneficial in avulsed teeth, which may also help in ITR [44,48,49,50]. Products such as Emdogain^®^ have shown promise in periodontal regeneration and reducing the risk of ankylosis in cases of ITR [48]. Emdogain^®^, an enamel matrix derivative, can serve as a cemental healer and promote regeneration of PDL, cementum, and alveolar bone [49]. Other root surface treatments such as sodium fluoride (NaF), ethylenediaminetetraacetic acid (EDTA), and liquid-phase concentrated growth factor (LPCGF) have also been introduced in the context of periodontal regeneration. In one case of delayed replantation, the use of NaF in the root surface treatment of an avulsed tooth was performed to delay ankylosis and decrease the rate of osseous replacement of the root surface [50]. Studies have demonstrated that the treatment of the root surface using EDTA and LPCGF together has increased the number of PDL cells adhering to a periodontally compromised root surface [44]. Though these studies have not been conducted in the context of ITR, these root surface treatments may be able to enhance the preservation and stimulation of PDL fibers while the tooth is treated extraorally. More research needs to be conducted in this area.

In the case of multi-rooted teeth, furcation areas and perforations into the furcation areas must be considered before replantation. In a study looking at 106 cases of ITR, 22.2% of cases had a perforation, and were indicated as the reason for carrying out ITR [51]. In the cases of perforation, use of a strong sealer to reduce bacterial contamination and promote healing is fundamental for a good prognosis. There have been reported cases of perforation into the furcation area as well as root perforations which have been repaired using sealers, such as MTA and calcium-enriched material (CEM) for perforation repair [52,53]. This involved removing any granulation tissue involved around the perforation site which was performed under excellent visualization due to the extraction of the tooth. Following granulation tissue removal, the area was prepared with ultrasonics and ultimately sealed with gray MTA. Following the procedure, the tooth appeared with radiolucency healing, no symptoms, and proper function [52]. Thus, ITR serves as an excellent option if perforation has occurred in the furcation as visibility is increased and direct sealing of the area and root resection, if needed, can occur. If no perforation is present in the furcation, it is important to remove any granulomatous tissue gently and examine them for structural integrity, especially if the tooth was sectioned.

## 6. Postoperative Stability

Postoperative stability of the replanted tooth comes down to the use of a splint as well as how the surrounding bone and periodontal microenvironment respond to the procedure. The use of a periodontal splint is recommended when teeth have short roots or there is significant mobility of class II or III [1]. When a multirooted tooth is sectioned or is surgically extracted, the socket wall must have adequate adaptation to the tooth root. Surgical manipulation may alter the ability for this adaptation to be strong, and splinting may be used to compensate for the lack of circumferential support. Some studies report using a splint for teeth that present with gross mobility, while other studies report using a splint for all cases [12]. Material selection varied as some clinicians used acrylic while others used flexible wires and sutures reducing rigid fixation and risk of ankylosis development [12]. However, splinting causes inability to conduct adequate oral hygiene and the patient must be educated on how best to keep areas clean. Studies have shown that the use of the splint can vary from 7 to 10 days to 3–4 weeks based on post-operative mobility, root length, and the clinician’s protocol [12,43]. Although evidence on splinting of intentionally replanted teeth that have been sectioned is limited, the operators need to make a decision that is clinically based on the relative stability of the treated sections.

Relief of the occlusal contact of the replanted tooth was key for reducing heavy functional stress during initial replantation and reintegration with the surrounding bone [12]. However, in most of these cases, occlusal adjustment was only performed when necessary and most teeth were replanted into proper functional occlusion [43].

With respect to teeth that required flap elevation, flaps can be repositioned and sutured to maximize healing and promote reconstruction of the periodontium [54]. Reducing the risk of inflammation at the root surface and alveolar socket is paramount in removing any likelihood of root resorption or ankylosis postoperatively.

## 7. Complications and Prognosis

The success of ITR and the long-term prognosis of the tooth highly depends on strict adherence to the protocols outlined here in addition to operator experience. Case selection, PDL preservation, minimization of PDL exposure time, and the degree of trauma when extracting all require meticulous technique by the endodontist and oral surgeon working in tandem. If any of these areas are not completed with adherence to ITR guidelines, the success of the procedure may be negatively impacted.

Tooth ankylosis, external root resorption, and replacement resorption are the most common complications following ITR and are examined during radiographic analysis postoperatively up to 2 years following the procedure [43]. To reduce these complications, limiting root surface damage is key to eliminate bone-to-tooth contact as well as limiting risk of infection postoperatively. Radiographically, following ITR, clinicians should observe radiolucency healing on radiograph, no post-operative symptoms such as pain, swelling, or functional sensitivity, and healing of the surrounding periodontal tissue [1]. From a procedural standpoint, all these marks of success can be attributed to proper atraumatic extraction technique, proper endodontic therapy, and maintenance of the tooth and socket.

## 8. Conclusions

This study aimed at categorizing the mandibular and maxillary dentition by their morphological characteristics while considering geographical and ethnic discrepancies that may exist. Additionally, surgical extraction techniques, such as sectioning and flap elevation, were discussed to help in creating extraction plans that may be feasible for complex teeth during ITR. Finally, management of the alveolar socket, root surface, and postoperative tooth stability are areas that could benefit from regenerative dental products. As biomaterials become increasingly advanced and their applications are improved, it will be important to see how these materials can improve procedural protocols. Understanding these aspects of ITR are crucial for both endodontist and oral surgeons and allows for both specialists to work in harmony during the procedure. While ITR remains a seldom used technique, clinicians should become aware of the techniques presented here to increase their scope and ability to perform the procedure. This would allow for more concrete data and support for success rates of ITR while considering the different procedural protocol. Further research needs to be conducted with regard to techniques such as root sectioning, flap elevation, and atraumatic extraction appliances, but the possible applications discussed may offer jumping off points for further discussion and investigation.

## Figures and Tables

**Table 1:** Root morphology characteristics by geographical location in the mandibular anterior dentition.

Study	Geographic Location	Number of Teeth Examined	Root Morphology Characteristics	Number of Roots	Mean Root Length
Malčić et al., 2006 [15]	Croatia	12,392	**Central Incisor:** 1.7% dilacerated **Lateral Incisor:** 0% dilacerated **Canine:** 1.2% dilacerated	All mandibular anterior dentition had 1 root	Not reported
Miloglu et al., 2010 [16]	Turkey	6386	**Central Incisor:** 0% dilacerated **Lateral Incisor:** 0% dilacerated **Canine:** 1.3% dilacerated	Not reported	Not reported
Sahebi et al., 2023 [17]	Iran	1537	**Central Incisors:** 4.9% dilacerated **Lateral Incisors**: 2.7% dilacerated **Canine:** 9.7% dilacerated Most frequent direction of dilaceration were distal, buccal, mesial, and lingual in that order 92.4% of dilacerations were mild (20–40°)	Not reported	Not reported
Tang et al., 2023 [18] Chen et al., 2023 [19]	China	106 [18] 4309 [19]	No dilaceration data reported. **Mandibular canines** and **lateral incisors** displayed root furcation: - 1% right canine, 1.5% for left canine, 0.2% for lateral incisors [16]	All incisors presented with one root	**Central Incisor:** 12.37 ± 1.24 mm **Lateral Incisor:** 11.09 ± 0.88 mm [15] **Right Central Incisor:** 12.22 ± 1.20 mm **Left Central Incisor:** 12.19 ±1.16 mm **Right Lateral Incisor:** 13.40 ± 1.27 mm **Left Lateral Incisor**: 13.36 ± 1.24 mm **Right Canine:** 15.40 ± 1.70 mm **Left Canine:** 15.55 ± 1.70 mm
Krishnan et al., 2024 [20]	India	400	**Central Incisors**: 3.5% distally curved, 0.5% buccally curved **Lateral Incisors**: 2.5% distally curved, 2% buccally curved, 0.5% mesially curved	**Central Incisors**: 100% one root **Lateral Incisors:** 100% one root	**Central Incisors**: 12.769 ± 1.128 mm **Lateral Incisors**: 13.044 ± 1.235 mm
Abdulsalam Ali Al-kinani et al., 2024 [21]	Yemen	192	Not reported	1.5% of canines had 2 roots (♂) 2.5% of canines had 2 roots in (♀)	**One rooted canine** (♂): 16.2 ± 1.6 mm **Two rooted canine** (♂): 11.8 ± 2.3 mm lingual and buccal root **One rooted canine** (♀): 14.8 ± 2.2 mm **Two rooted canine** (♀): 13.3 ± 0.4 mm

**♂**: signifies male population; **♀**: signifies female population.

**Table 2:** Root morphology characteristics by geographical location in the maxillary anterior dentition.

Study	Geographic Location	Number of Teeth Examined	Root Morphology Characteristics	Number of Roots	Mean Root Length
Udoye et al., 2009 [22]	Nigeria	706	**Central Incisor:** 0% dilacerated **Lateral Incisor:** 2.5% dilacerated **Canine:** 0% dilacerated * Females had greater predilections to dilacerations	Not reported	Not reported
Bernardes et al., 2021 [23]	Brazil	400	Apical root morphology **central Incisor**: 6.5% short, 49.5% blunt (rhomboid), 9.5% curved, 34.5% pipette shaped Apical root morphology **lateral incisor**: 2.5% short, 23.5% blunt (rhomboid), 55.5% curved, 18.5% pipette shaped [23]	Not reported	Not reported
Sahebi et al., 2023 [17]	Iran	1537	**Central Incisor:** 1.5% dilacerated **Lateral Incisor:** 7.1% dilacerated **Canine:** 9.8% dilacerated - 92.4% of dilacerations were mild (20–40°) and 83.7% occurred in the apical 1/3 of the tooth root	Not reported	Not reported
Chen et al., 2023 [19]	China	4309	No furcation observed in maxillary anterior teeth.	All maxillary anterior teeth had 1 root	**Right Central Incisor:** 13.39 ± 1.69 mm **Left Central Incisor:** 13.32 ± 1.74 mm **Right Lateral Incisor:** 13.48 ± 1.54 mm **Left Lateral Incisor:** 13.32 ± 1.74 mm **Right Canine:** 16.78 ± 1.94 mm **Left Canine:** 16.54 ± 2.11 mm
Hasan et al., 2023 [24]	Iraq	389 patients where full dentition was examined	**Central Incisor:** 14.1% dilacerated, 100% of dilaceration was in apical 1/3 **Lateral Incisor:** 40.2% dilacerated, 67.5% of dilaceration was in apical 1/3, 32.5% in middle third **Canine:** 26.1% dilacerated, 100% of dilaceration in apical 1/3	Not reported	Not reported
El Sheikh et al., 2025 [25]	Egypt	600 patients where full dentition was examined	Not reported	Not reported	**Right Canine:** ♂: 14.28 ± 0.388 mm ♀: 13.98 ± 0.356 mm **Left Canine:** ♂: 14.05 ± 0.366 mm ♀: 14.03 ± 0.368 mm **Left Lateral Incisor:** ♂: 12.02 ± 0.215 mm ♀: 12.05 ± 0.184 mm **Right Lateral Incisor:** ♂: 12.32 ± 0.220 mm ♀: 12.17 ± 0.163 mm **Right Central Incisor:** ♂: 14.11 ± 0.465 mm ♀: 14.05 ± 0.384 mm **Left Central Incisor:** ♂: 14.37 ± 0.585 mm ♀: 13.94 ± 0.392 mm

**♂**: signifies male population; **♀**: signifies female population.

**Table 3:** Root morphology characteristics by geographical location in the mandibular posterior dentition.

Study	Geographic Location	Number of Teeth Examined	Root Morphology Characteristics	Number of Roots
Llena et al., 2014 [26]	Spain	126	Mean length of **first and second premolar** roots in males was 16.05 mm and 14.91 mm in females (significant difference) **First premolar**: 34.2% had no root angulation, 9.6% had buccal angulation, 12.3% had lingual angulation, 13.7% had mesial angulation, and 30.1% had distal angulation **Second premolar**: 45.3% had no root angulation, 22.6% had buccal angulation, 7.5% had lingual angulation, 7.5% had mesial angulation, 7.0% had distal angulation	All mandibular premolars were single rooted
Felsypremila et al., 2015 [27]	India	3015	Bilateral anatomic symmetry was seen in 96.1% of **mandibular first premolars** 98.3% of **mandibular second premolars** had bilateral anatomic symmetry Bilateral anatomic symmetry was seen in 78.6% of **mandibular first molars** 82.1% of **mandibular second molars** had bilateral anatomic symmetry	**Mandibular first premolar**: 2% had 2 roots, 98% had 1 root **Mandibular second premolar:** 99.7% had 1 root, 0.3% had 2 roots **Mandibular first molar:** 5.7% had 3 roots, 93.6% had 2 roots, 0.7% had 1 root **Mandibular second molar:** 2.5% had 3 roots, 88.8% had 2 roots, and 8.7% had 1 root
Asheghi et al., 2022 [28]	Iran	472	20.0% of **mandibular first molars** had dilaceration 15.3% of **mandibular second molars** had dilaceration	Not reported
Hasan et al., 2023 [24]	Iraq	389 patients where full dentition was examined	**First premolar:** 0.0% dilaceration **Second premolar:** 6.8% dilaceration **First molar**: 6.8% dilaceration **Second molar:** 6.8% **Second molars** had 66.7% of dilacerations occurring in middle 1/3 and 33.3% in the apical 1/3	Not reported
Tang et al., 2025 [29]	China	110	Average root length of 3 rooted **first molars**: 9.17 ± 1.40 mm Average root length of 2 rooted **first molars:** 9.68 ± 1.31 mm Average root length of 2 rooted **second molars**: 8.85 ± 1.37 mm All mesial roots curved severely (81.8%) or moderately (18.2%) towards the furcation side No straight roots observed All mesial roots except one exhibited distal concavities Mesial concavities only observed in 76.0% of first molars and 60% of second molars	67% of **mandibular first molars** had 2 roots 33% of **mandibular first molars** had 3 roots 100% of **mandibular second molars** had 2 roots
Pataer et al., 2025 [30]	China	1748 patients, specifically looking at patients with C-shape canal morphology	12.8% of **second molars** had two separate divergent/parallel roots with clear trabeculae between them 40.5% of **second molars** had two separate converging roots with clear trabeculae between them Longitudinal grooves mainly located on the lingual surface	10.2% of **mandibular second molars** had 1 root 34.7% of **mandibular second molars** had 2 roots 52.1% of **mandibular second molars** had 3 roots 3.0% of **mandibular second molars** had 4 roots

**Table 4:** Root morphology characteristics by geographical location in the maxillary posterior dentition.

Study	Geographic Location	Number of Teeth Examined	Root Morphology Characteristics	Number of Roots
Miloglu et al., 2010 [16]	Turkey	6386	**First Premolar:** 3.2% root dilaceration **Second Premolar**: 5.1% root dilaceration **First Molar:** 6.7% root dilaceration **Second molar:** 5.4% root dilaceration	Not reported
Felsypremila et al., 2015 [27]	India	3015	Tooth anatomic symmetry was seen in 81.5% of **maxillary first premolars** and 81.5% of **maxillary second premolars** Tooth anatomic symmetry was seen in 77.5% of **mandibular first molars** and 70.8% of **mandibular second molars**	**Maxillary first premolar:** 51.2% had 2 roots, 48.8% had 1 root **Maxillary second premolar:** 90.6% had 1 root, 9.4% had 2 roots **Maxillary first molar:** 0.5% had 4 roots, 96.8% had 3 roots, 2.7% had 2 roots **Maxillary second molar**: 1.1% had 4 roots, 80.3% had 3 roots, 8.9% had 2 roots, and 9.7% had 1 root
Marcano-Caldera et al., 2019 [31]	Colombia	1359 CBCT scans	43.2% of **maxillary molars** presented with some type of radicular fusion Root fusion was observed in 23.3% of **first maxillary molars**, 57.7% of second maxillary molars Type 3 fusion (DB root fused with palatal root) was most common in **first maxillary molars** Type 6 fusion (P, MB, and DB roots fused as a cone shaped root) was most common in **second maxillary molars**	Not reported
Qiao et al., 2021 [32]	China	274	Mean curvature of **maxillary second premolar** was higher than that of **first premolar**. All premolars had moderate curvature (5–20°) in mesiodistal and buccolingual directions MB1 and MB2 of **maxillary first molars** and MB2 of maxillary second molars showed severe bending (>20°) in mesiodistal directions	Not reported
Yan et al., 2021 [33]	China	1118	56.4% if **maxillary second premolars** were curved. 44.2% of **maxillary second premolars** were moderately curved (10–25°) mesiodistally and 12.2% were severely curved mesiodistally (>25°) 29.5% of **maxillary second premolars** were also moderately curved buccopalatally and 11.4% were severely curved buccopalatally The mean distance from the root tip to the floor of the maxillary sinus was 2.47 ± 3.45 mm	94.2% of **maxillary second premolars** had 1 root 5.8% of **maxillary second premolars** had 2 roots
Hasan et al., 2023 [24]	Iraq	389 patients where full dentition was examined	**First premolar:** 6.5% dilaceration **Second premolar**: 0% dilaceration **First molar:** 0% dilaceration **Second molar:** 13.1% **Second molars** had 66.7% of dilacerations occurring in middle 1/3 and 33.3% in the apical 1/3	Not reported

## Data Availability

All data supporting results and findings are reported here.
